# The Role of Vitamins and Micronutrients in the Prevention of Melanoma: A Review of Current Evidence

**DOI:** 10.3390/ijms27031428

**Published:** 2026-01-31

**Authors:** Joanna Pec, Weronika Pająk, Jakub Kleinrok, Kamil Rusztyn, Jolanta Flieger, Barbara Teresińska, Alicja Forma, Jacek Baj

**Affiliations:** 1 Chair and Department of Forensic Medicine, Medical University of Lublin, Ul. Jaczewskiego 8b, 20-090 Lublin, Polandwapajak@gmail.com (W.P.); klejs.90@gmail.com (J.K.);; 2 Faculty of Medicine, Medical University of Warsaw, Ul. Żwirki i Wigury 61, 02-091 Warsaw, Poland; kamil.rusztyn12@gmail.com; 3 Department of Analytical Chemistry, Medical University of Lublin, Chodźki 4a (Collegium Pharmaceuticum), 20-093 Lublin, Poland; jolanta.flieger@umlub.edu.pl; 4 Doctoral School, Medical University of Lublin, Chodźki 7, 20-093 Lublin, Poland; 5 Department of Correct, Clinical, and Imaging Anatomy, Medical University of Lublin, Jaczewskiego 4, 20-090 Lublin, Poland

**Keywords:** melanoma, prevention, nutrition, oxidative stress, micronutrients, vitamins

## Abstract

Melanoma is a type of skin cancer with an increasing incidence rate worldwide and a high mortality rate. In addition to known risk factors, such as UV exposure and genetic predisposition, researchers are paying more attention to the role of diet, micronutrients, and vitamins in preventing melanoma. This review discusses the effects of selected vitamins (D, A, C, and E), trace elements, and bioactive compounds (polyphenols and omega-3 fatty acids) on biological processes related to melanoma development. The review considered both antioxidant and immunomodulatory effects, as well as effects on DNA repair and photoprotection. The significance of polymorphisms of genes encoding receptors and enzymes that metabolize the compounds studied was also analyzed. The results suggest that maintaining adequate levels of these substances may promote melanoma prevention, particularly among individuals at risk. However, caution in the use of supplementation is necessary due to the possible biphasic effects of some micronutrients. Further clinical trials are needed to develop effective, safe prevention strategies based on micronutrients and vitamins.

## 1. Introduction

Melanoma is a type of skin cancer that originates from specialized cells called melanocytes. Its incidence is steadily increasing worldwide. As recently as 1935, the estimated risk of developing melanoma was approximately 1 in 500 people. Currently, this risk has increased significantly, and it is estimated to be 1 in 50 for the Western population. What is more, according to forecasts, by 2040 we can expect another increase in melanoma cases, by up to 50%, which equates to 510,000 new cases and thus an increase in deaths from the disease. Despite the substantial increase in incidence, melanoma is not the most prevalent form of skin cancer at this point. However, it is important to note that it does have the highest mortality rate. This type of cancer is responsible for nearly 73% of deaths among patients diagnosed with skin cancer. Melanoma development is influenced by a combination of environmental and genetic factors [1,2,3]. A positive family history and certain phenotypic characteristics, such as fair skin or red hair, increase a patient’s risk of melanoma. Patients with certain genetic diseases, such as xeroderma pigmentosum, or with genetic mutations, such as CDK2NA or CDK4, are at significant risk of melanoma [4]. Environmental factors are equally important, particularly the patient’s exposure to ultraviolet (UV) radiation, which exhibits genotoxic effects. It is assumed that 60–70% of skin melanomas are caused by UV exposure [5].

In the prevention of melanoma, in addition to avoiding excessive sun exposure or tanning bed use, the influence of diet and supplementation seems to be important. Studies show that a diet rich in specific nutrients or supplementation with them can positively impact the immune system and reduce the risk of melanoma [5,6]. The effects of micronutrients, including selenium, zinc, and copper, were discussed. These micronutrients have anti-inflammatory and antioxidant properties that may lower melanoma risk in the general population. The influence of certain vitamins, such as vitamins D, A, C, and E, seems equally important [3,7]. Other bioactive dietary components, such as polyphenols and omega-3 fatty acids, may also be worth considering melanoma prevention [6].

The increasing incidence of melanoma cases in the population underscores the necessity for effective disease prevention strategies. A potential strategy for melanoma prevention, particularly among individuals at risk, involves dietary intervention and supplementation [3].

## 2. Search Strategy and Aim of the Paper

The aim of this review is to evaluate the current evidence on the role of selected micronutrients and vitamins in the prevention of melanoma. Given the increasing global incidence of melanoma [8] and the emerging understanding of its oxidative and immunological fundamentals, particular attention is paid to compounds with antioxidant, immunomodulatory, and photoprotective properties. This paper focuses on vitamins D, A, C, and E, as well as trace elements such as selenium and zinc, discussing their mechanisms of action and clinical relevance. These vitamins were pre-specified based on (i) their recurring presence in the melanoma carcinogenesis literature, (ii) their relevance to UV-related pathways highlighted in this review, and (iii) the availability of clinically interpretable human data. The search strategy also captured other essential vitamins, however. However, where melanoma-specific evidence was limited or heterogeneous, these were not assigned dedicated subsections and are only briefly addressed as evidence group.

A comprehensive literature search was conducted using PubMed, Scopus, and Web of Science databases, covering publications from January 2010 to April 2025. The following keywords and Boolean operators were used: (“melanoma”) AND (“vitamins” OR “vitamin D” OR “vitamin A” OR “vitamin C” OR “ascorbic acid” OR “vitamin E” OR “selenium” OR “zinc” OR “micronutrients” OR “copper” OR “manganese” OR “iron” OR “fluorine” OR “iodine” OR “chromium” OR “molybdenum” OR “polyphenols” OR “omega-3 fatty acids”) AND (“prevention” OR “epidemiology” OR “risk factors” OR “risk reduction” OR “oxidative stress” OR “UV damage” OR “nutrition”).

Inclusion criteria were:

- Original articles, reviews, meta-analyses, and clinical trials published in peer-reviewed journals;
- Studies addressing molecular mechanisms, clinical correlations, or public health implications;
- English-language publications.

Exclusion criteria included:

- Non-melanoma skin cancers, unless explicitly related to shared mechanisms;
- Studies lacking relevance to the preventive or protective roles of micronutrients;
- In vitro studies without translational implications.

This review integrates findings from experimental, clinical, and epidemiological research to present a multidisciplinary overview of micronutrient-based strategies for melanoma prevention.

## 3. Oxidative Stress and UV-Induced DNA Damage in Melanomagenesis

UV radiation is a crucial environmental factor in melanomagenesis, which initiates genetic alterations via direct DNA damage and secondary oxidative pathways. UVB (280–315 nm) is mainly responsible for direct DNA lesions, particularly cyclobutane pyrimidine dimers (CPDs) and 6–4 photoproducts. On the other hand, UVA (315–400 nm) penetrates deeper into the skin and primarily induces oxidative stress by photosensitizating endogenous chromophores [9]. This leads to the formation of reactive oxygen species (ROS) and reactive nitrogen species (RNS), including superoxide anion (O_2_•^−^), hydrogen peroxide (H_2_O_2_), hydroxyl radicals (•OH), and nitric oxide (NO), which do damage to the nucleic acids, proteins, and lipids [10,11]. Melanocytes are particularly sensitive to oxidative stress because they generate ROS during melanin synthesis. Melanosomes, organelles in which melanin is created, contribute to intracellular oxidative load, especially when exposed to UV radiation [10]. Notably, pheomelanin—a red-yellow pigment predominant in individuals with lighter skin—exhibits prooxidant properties and enhances UVA-induced ROS generation, while eumelanin (present in a higher rate in individuals with darker skin) acts as a protective antioxidant [12]. A growing body of evidence suggests that oxidative stress impairs DNA repair mechanisms, particularly nucleotide excision repair (NER), which is essential for removing UV-induced DNA lesions. UVA exposure has been shown to degrade key repair proteins such as PCNA and RPA, resulting in delayed repair of CPDs [13]. Furthermore, UVA-induced “dark CPDs”—mutagenic lesions that form hours after exposure due to melanin-dependent chemiexcitation—extend the genotoxic impact of UV beyond the irradiation period [13]. To counteract oxidative damage, skin cells activate cytoprotective responses such as the Nrf2 (nuclear factor erythroid 2–related factor 2) signaling pathway, which upregulates genes encoding antioxidant and detoxifying enzymes such as HO-1, NQO1, and glutathione-related proteins [14,15]. However, the efficiency of this defence varies between cell types and can be insufficient under chronic UV exposure. In addition, epigenetic mechanisms and microRNA (miRNA) regulation are modulated by ROS, promoting malignant transformation. To be more specified, oxidative stress leads to the downregulation of tumor-suppressive miRNAs (e.g., miR-125b) via DNA hypermethylation, while activating oncogenic miRNAs such as miR-21 through redox-sensitive transcription factors (e.g., STAT3, NF-κB) [11]. Also, genetic predispositions influence individual susceptibility to UV-induced melanomagenesis. Polymorphisms in genes encoding antioxidant enzymes (e.g., CAT, NOS1) or pigmentation regulators (e.g., MC1R) modulate how efficiently the skin handles oxidative stress and DNA damage following sun exposure [15]. These gene–environment interactions (GxE) highlight the complexity of UV-induced melanoma risk and highlight the importance of personalized prevention strategies.

## 4. Vitamins and Melanoma

### 4.1. Vitamin D

#### 4.1.1. Sources, Skin Synthesis, and Metabolism

**Figure 1:**
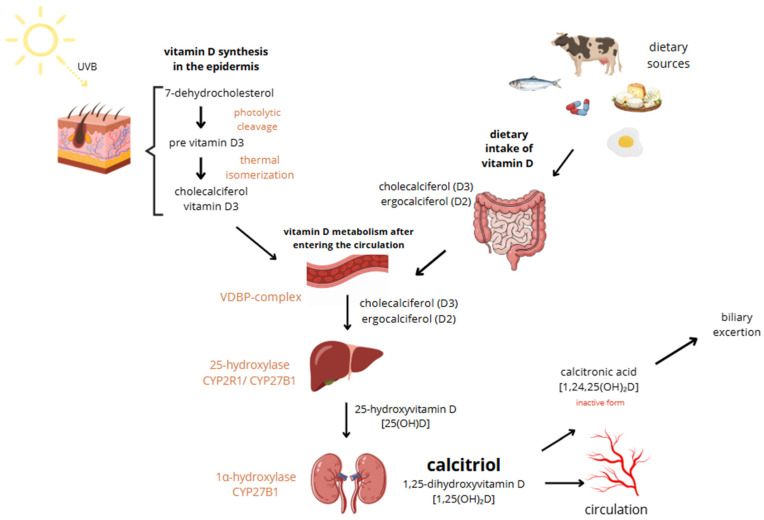
Skin synthesis and metabolism of vitamin D.

Vitamin D, a fat-soluble vitamin essential for calcium absorption, bone mineral regulation, and muscle function, exists in two main forms: vitamin D2 (ergocalciferol) and vitamin D3 (cholecalciferol). While D2 is derived from plant sources and added to foods due to the body’s inability to synthesize it, D3 is produced in the skin through sunlight exposure, making cutaneous synthesis its main source. However, it can also be obtained from certain foods such as fish, beef liver, egg yolk, and cheese, as well as from supplements. Both vitamin D3 oral intake and sun exposure aim to achieve relevant vitamin D levels. Vitamin D3 is more potent than D2 and is typically preferred for treating deficiencies, used as a supplement [16]. It is said that the Earth’s population generally has low vitamin D levels [17]. Sun exposure up to about 25% of the skin surface—such as the face, hands, and arms—at a quarter of the minimum erythemal dose (MED) can generate approximately 1000 IU of vitamin D, while full-body exposure for 15 min at midday in summer may yield up to 10,000 IU. However, the efficiency of vitamin D synthesis varies widely depending on factors such as age, skin pigmentation, time of day, geographic location, weather, season, clothing, and sunscreen use [18]. In old age, cutaneous synthesis of vitamin D can be reduced to as little as 25%. Combined with a decline in the kidneys’ ability to hydroxylate vitamin D, this can lead to significant deficiencies [19]. The great majority of vitamin D3 is synthesized endogenously in the epidermal keratinocytes following exposure to ultraviolet B (UVB) radiation. It induces photolytic cleavage of 7-dehydrocholesterol to pre-vitamin D3. This unstable intermediate undergoes thermal isomerization to form cholecalciferol, which then enters circulation bound to vitamin D-binding protein (VDBP). Excessive vitamin D synthesis is self-limited by conversion of pre-vitamin D3 to inactive photoproducts (e.g., lumisterol, tachysterol), preventing vitamin D toxicity from sun exposure [20]. In circulation, vitamin D is hydroxylated in the liver to form 25-hydroxyvitamin D [25(OH)D] in mediation of CYP2R1 or CYP27B1. The major circulating metabolite has a half-life of 2–3 weeks. Subsequent hydroxylation occurs primarily in the renal proximal and distal tubules via 1α-hydroxylase (CYP27B1), producing the biologically active form, 1,25-dihydroxyvitamin D [1,25(OH)_2_D or calcitriol], which has a short half-life of 4–8 h [19,21]. Calcitriol exerts its effects through the intracellular vitamin D receptor (VDR), a nuclear receptor expressed in multiple tissues, including immune, bone, intestinal, kidney, and epithelial cells. Upon ligand binding, VDR heterodimerizes with the retinoid X receptor and binds to vitamin D response elements (VDREs) in target genes, modulating their transcription. The above-mentioned mechanisms lead to the inhibition of cellular proliferation, enhancement of cellular differentiation, modulation of immune activity, regulation of ROS levels, and alterations in mitochondrial function [19]. In the non-genomic pathway, vitamin D elicits rapid cellular responses by binding to membrane-associated receptor proteins such as PDIA3 (also known as 1,25D3-MARRS), which is located on both the endoplasmic reticulum and cytoplasmic sides. PDIA3 facilitates signaling cascades involved in calcium and phosphate uptake, protein folding, and modulation of second messenger systems (IP3, DAG, cAMP), ultimately contributing to the rapid activation of pathways like WNT5A [19]. The activation and degradation of vitamin D metabolites are tightly regulated by parathyroid hormone (PTH), calcium, phosphorus, and fibroblast growth factor 23 (FGF23). FGF23 downregulates renal CYP27B1 activity and enhances expression of 24-hydroxylase (CYP24A1), promoting catabolism of both 25(OH)D and 1,25(OH)_2_D. Magnesium serves as a critical cofactor in several enzymatic steps of vitamin D metabolism, including hepatic and renal hydroxylation, and its deficiency impairs both the bioactivation and transport of vitamin D. This reciprocal relationship between magnesium and vitamin D deficiencies may contribute to adverse clinical outcomes, such as an increased risk of fragility fractures, particularly in postmenopausal women. Extra-renal expression of CYP27B1 in tissues such as skin, breast, and macrophages can lead to ectopic production of calcitriol, contributing to pathological hypercalcemia in granulomatous diseases like sarcoidosis [19,22]. Skin synthesis and metabolism of vitamin D are shown in Figure 1.

#### 4.1.2. Immunomodulatory Effects

Vitamin D plays a crucial role in immune system regulation by influencing suppressor T lymphocyte activity, controlling cytokine production, and modulating cellular apoptosis processes [23]. Its role in the innate immune system is played by an active form, 1,25-dihydroxyvitamin D [1,25(OH)_2_D], synthesized locally by macrophages and monocytes upon activation by toll-like receptor (TLR) signaling and pro-inflammatory cytokines such as interferon-γ (IFN-γ). This intracrine signaling enhances antimicrobial responses via upregulating the expression of the enzyme CYP27B1 and VDR activation, leading to upregulation of cathelicidin (LL-37) and β-defensins, peptides with broad-spectrum antimicrobial and antiviral activity [24]. Additionally, vitamin D also modulates the phenotype and function of antigen-presenting cells (APCs), such as dendritic cells and monocyte-derived macrophages. 1,25(OH)_2_D downregulates the expression of major histocompatibility complex (MHC) class II and co-stimulatory molecules (CD80, CD86), leading to a tolerogenic APC phenotype with diminished capacity to activate naive T cells. This is associated with reduced production of IL-12 and increased synthesis of the anti-inflammatory cytokine IL-10. Furthermore, vitamin D decreases TLR expression on monocytes and suppresses the production of pro-inflammatory cytokines, including IL-6, IL-17, and IL-2, which are key mediators in chronic inflammation and autoimmunity.

It also influences endothelial stability, nitric oxide production, and reduces vascular permeability through genomic and rapid non-genomic mechanisms. It upregulates endothelial nitric oxide synthase (eNOS) gene expression, enhancing NO production, which is critical for vascular dilation and protection against oxidative stress. Non-genomic actions involve rapid activation of second messenger systems, including cyclic adenosine monophosphate (cAMP) and inositol trisphosphate/diacylglycerol (IP_3_/DAG), and calcium mobilization [21,25]. In the intestinal epithelium, vitamin D supports the integrity of the mucosal and epithelial barriers. It promotes the expression of tight junction proteins (e.g., occludin, claudins), reduces epithelial apoptosis, and stimulates the production of antimicrobial peptides by Paneth cells and intraepithelial lymphocytes. Therefore, vitamin D helps prevent microbial translocation and supports immune tolerance to commensal microbiota [26]. Within the adaptive immune system, intracrinely synthesized 1,25(OH)_2_D, upon activation of VDR and CYP27B1, shifts T-cell responses from proinflammatory TH1 (IL-2, IFN-c, TNF-a) and TH17 (IL 17, IL-21) cytokines to anti-inflammatory TH2 (IL-4, IL-5, IL-9, IL-13) and Treg phenotypes. On the contrary, activated VDR further inhibits B-cell proliferation into plasma cells and autoantibody production while promoting B-cell apoptosis. 1,25(OH)_2_D also enhances the secretion of anti-inflammatory cytokines such as IL-10 and CCR10. These effects collectively reduce the risk of autoantibody-mediated diseases [27,28]. Moreover, vitamin D deficiency is strongly associated with subclinical inflammation. It is regulated by the innate immune system through pathways involving nuclear factor kappa B (NFκB), activator protein-1 (AP-1), and their anti-inflammatory counterparts PPAR-γ. Pro-inflammatory cytokines such as TNF-α and IL-6, along with biomarkers including hs-CRP, ferritin, albumin, and neutrophil-to-lymphocyte ratio (NLR), are commonly elevated [22,29].

#### 4.1.3. Evidence Linking Vitamin D Levels and Melanoma Risk

The origin of melanoma has been greatly researched, stating that prolonged sun exposure and genetics are two of the risk factors for its occurrence. Melanoma can appear anywhere within human organisms, including organs other than the skin. Vitamin D is believed to play an antiproliferative and preventive role in melanoma. VDR plays a key role in limiting tumor growth by suppressing the proliferation of cancer cells and promoting their apoptosis. It also influences important cellular functions such as interactions with growth factors, cell adhesion, metastasis, and autophagy. Therefore, VDR is considered to act as a tumor suppressor, including in cancers caused by UV radiation [30]. We can distinguish UVA and UVB radiations, both carcinogenic, inducing DNA damage, which leads to mutations and further cancer transformation. UVA cannot be blocked by external materials. Therefore, it penetrates deeply into the dermal layers. It is the key cause of photoaging by DNA damage, collagen, and elastin fibers degradation through oxidative stress pathways. On the other hand, UVB is primarily absorbed by the outer layer of the skin, epidermis, playing a vital and unique role in vitamin D3 cutaneous synthesis. UVA and UVB both cause cell inflammation and breakdown of the extracellular matrix (ECM) proteins. Cells can be protected from the carcinogenic effects of UV radiation by vitamin D3. It protects the tissues from photodamage by repairing CPDs, reducing oxidative stress, and chronic inflammation [31]. Moreover, 1,25(OH)_2_D_3_ supports the health of various skin cell types—including keratinocytes, melanocytes, and fibroblasts—when they are exposed to UV radiation. Interestingly, both 1,25(OH)_2_D_3_ and its synthetic analogs have been found to prevent UVB-induced cell death by enhancing DNA repair mechanisms, which in turn reduces the need for apoptosis. Additionally, 1,25(OH)_2_D_3_ can suppress the production of the inflammatory cytokine interleukin-6 (IL-6) in keratinocytes. Altogether, these actions highlight the protective role of 1,25(OH)_2_D_3_ in limiting UV-induced cellular damage and inflammation [32]. A 2023 cross-sectional study in 498 adults found that regular 1,25(OH)_2_D_3_ oral intake can reduce the incidence of melanoma and other skin cancers compared to non-users. The regular supplementation correlated positively with a lower percentage of past or present melanoma history. However, it did not affect the photoaging of the skin [33]. Multiple metabolites of vitamin D_3_ and lumisterol exhibit diverse biological activities through genomic and non-genomic mechanisms mediated by nuclear receptors [VDR, retinoic acid-related orphan receptors (RORα/γ), liver X receptors (LXRα/β), aryl hydrocarbon receptor (AhR), and peroxisome proliferator-activated receptor γ (PPARγ)]. Several of these metabolites, particularly those lacking calcemic activity, show selective receptor activation profiles and exert anticancer, anti-inflammatory, and antifibrotic effects. Hydroxylumisterols and hydroxyvitamin D derivatives also provide photoprotection in melanocytes by reducing UV-induced DNA damage via upregulation of tumor suppressors (p53, PTEN) and enhancement of DNA repair mechanisms (XPC expression). Importantly, these compounds inhibit melanoma proliferation while preserving or minimally affecting normal melanocyte function, suggesting a therapeutic potential in melanoma prevention and treatment [34]. However, dysregulation of vitamin D metabolism may impair the anticancer efficacy of active vitamin D forms and is associated with poor melanoma prognosis. Clinical studies have suggested that vitamin D supplementation, particularly when combined with calcium, may reduce melanoma risk, especially in individuals with a history of skin cancer [35,36]. A 2024 study focused on the possible therapeutic approach of the novel FGFR inhibitor in melanoma treatment, given the high expression and mutation rate of FGFRs in this cancer. Using primary and metastatic melanoma cell lines, the inhibitor showed significant antiproliferative effects. Co-treatment with active vitamin D further enhanced its efficacy. The inhibitor induced G_0_/G_1_ cell cycle arrest and apoptosis, with vitamin D modulating these effects. Mechanistically, 1,25(OH)_2_D_3_ decreased FGFR1/2 protein levels and activation in cells, potentially explaining the enhanced sensitivity. These results support further exploration of the inhibitor, especially in combination with vitamin D, as a targeted strategy in FGFR-expressing melanomas [37]. A 2021 randomized, placebo-controlled trial evaluated the impact of vitamin D supplementation on melanoma outcomes. Patients with stage II melanoma received 100,000 IU of vitamin D every 50 days for three years. While the supplementation was safe and well tolerated, individuals with Breslow thickness ≥ 3 mm at diagnosis showed a lower increase in circulating 25OHD levels and were more likely to relapse, suggesting impaired vitamin D responsiveness potentially linked to reduced expression of vitamin D-related proteins. However, VDR polymorphisms, particularly the BsmI B allele, showed a potential protective effect, aligning with previous findings. The study results support a role for vitamin D in melanoma prognosis. It may be a useful preventive strategy in low-risk melanoma patients, whereas high-risk patients could benefit from combination approaches involving immunomodulation [38]. Many studies confirmed vitamin D’s contribution to the protection against DNA damage, immune suppression, and the development of skin cancers. In the 2022 study, scientists highlighted that treatment with vitamin D significantly decreased the viability of human melanoma cell lines and elevated caspase activity, indicative of apoptosis. These effects were absent in cell lines deficient in both the VDR and the tumor suppressor PTEN, which is commonly lost or mutated in melanoma. Exposure to 1,25D led to a marked increase in PTEN expression and inhibition of the AKT signaling pathway, along with its downstream targets. These findings suggest that 1,25D may suppress melanoma cell survival by modulating PTEN expression and inhibiting AKT-driven oncogenic signaling [39]. Furthermore, beyond its role in supporting the immune system, vitamin D is also recognized for its anticancer properties. These include its ability to slow cell proliferation, trigger programmed cell death (apoptosis), prevent the spread and invasion of cancer cells, inhibit metastasis formation, and reduce the development of new blood vessels (antiangiogenic effects) [40]. A 2023 study demonstrated that the incorporation of anti-PD-1 antibodies has significantly improved outcomes for patients with advanced melanoma. This study suggests that normal serum vitamin D levels are associated with improved objective response rates (ORR) (56%) and median PFS (11.25 months), compared to patients with suboptimal vitamin D levels. The biological plausibility of vitamin D’s impact on immunotherapy efficacy is supported by its immunomodulatory and anticancer effects, including enhanced T-cell activity, inhibition of inflammatory cytokines, and modulation of tumor microenvironment infiltration. Furthermore, vitamin D may reduce immune-related adverse events and influence PD-L1 expression, potentially enhancing response to checkpoint inhibitors [41]. Vitamin D deficiency is not only a risk factor for melanoma, but also for non-melanoma skin cancers [42]. A 2023 study states that sun exposure, though essential for vitamin D production, is not correlated with sunny regions, such as Greece. Among non-melanoma skin cancer patients, despite abundant UV exposure, vitamin D levels did not consistently correlate with skin cancer risk, indicating a complex relationship likely influenced by additional factors such as oxidative stress. Higher vitamin D levels were associated with better redox profiles, suggesting a protective antioxidant role for vitamin D in maintaining cellular homeostasis [43].

#### 4.1.4. VDR Polymorphisms and Skin Cancer Susceptibility

The VDR gene, located on chromosome 12q13.11, encodes a nuclear receptor involved in mediating the effects of 1,25-dihydroxyvitamin D_3_ on gene expression. Structurally, VDR belongs to the steroid and thyroid hormone receptor superfamily and comprises distinct functional domains for DNA binding, ligand binding, and transcriptional activation. It regulates a wide array of physiological processes, including calcium–phosphate homeostasis, cell proliferation, differentiation, and immune modulation [44]. In a great variety of recent research, certain genetic variations in the VDR gene have been associated with the risk and development of malignant melanoma [30]. VDR expression appears to inversely correlate with tumor progression, with higher levels typically found in noncancerous or early-stage tissues and reduced or absent expression associated with malignant transformation [45]. Previous studies established that reduced expression of VDR in the cytoplasm and nucleus is associated with malignant progression of cutaneous melanoma (CM). It was suggested that a genetic factor may play a role in VDR expression patterns. Significant findings included a gender-related reduction in cytoplasmic VDR expression in female patients and increased cytoplasmic VDR levels associated with vitamin D supplementation. High sun exposure and the presence of actinic keratosis were also linked with elevated cytoplasmic VDR expression, whereas idiopathic guttate hypomelanosis correlated with reduced levels. A reduction in nuclear VDR was observed in patients with fair skin and lighter hair colors. Multivariable analysis confirmed only high sun exposure as significantly associated with increased cytoplasmic VDR expression [45,46]. In healthy skin, VDR and retinoid X receptor (RXRα) in keratinocytes and melanocytes modulate UV-induced damage responses, proliferation, and immune surveillance. Loss or cytoplasmic mislocalization of these receptors disrupts melanocyte–keratinocyte crosstalk, enhances Wnt/β-catenin signaling, and promotes melanoma initiation and progression [44,47,48]. Furthermore, studies showed that VDR gene polymorphisms (FokI, ApaI, BsmI, and TaqI) are associated with altered melanoma risk [49,50,51]. These polymorphisms can influence mRNA stability, protein structure, and transcriptional activity, potentially altering disease susceptibility. For instance, the FokI polymorphism affects the translation initiation site, producing a shorter or full-length VDR protein. Other SNPs, such as BsmI, ApaI, and TaqI, are located near or within non-coding regions and can impact gene expression through regulatory mechanisms. Variants in the VDR gene are associated with increased risk for various chronic and immune-mediated conditions, highlighting their clinical relevance in personalized medicine and disease prevention strategies [52]. A 2025 study showed that the FokI CT genotype is linked to an increased risk of melanoma, whereas the BsmI AG genotype is protective. For non-melanoma skin cancers (NMSC), the FokI TT genotype is a risk factor, and the CC genotype is protective; similarly, the TaqI T allele and TT genotype have protective effects. These associations vary by geographic region and study period, suggesting environmental and temporal influences on VDR-related skin cancer susceptibility [53]. Moreover, VDR agonists are placed as potential therapeutic agents for vitiligo. The VDR gene plays a crucial role in mediating vitamin D’s immunoregulatory effects, and missense mutations may impair its function, contributing to vitiligo pathogenesis [54]. Another 2025 study highlights potential molecular targets—VDR/FOK1, CYP27A1/CYP27B1, and CYP2R1/CYP24A1—for diagnostic and therapeutic development. It was discovered that the absence of those CYP450 enzymes correlates with pronounced VitD_3_-mediated growth inhibition, suggesting reduced diversion of VitD through alternative pathways [55].

#### 4.1.5. Supplementation and Sun Exposure Balance

A 2024 prospective, randomized, double-blind, placebo-controlled trial was conducted to assess whether high-dose vitamin D supplementation could reduce relapse in patients with resected CM, stages IA to III. A total of 436 patients were randomized to receive either monthly 100,000 IU of cholecalciferol or placebo for up to 42 months, with a median follow-up of 52 months. Vitamin D supplementation significantly increased serum 25-hydroxyvitamin D levels without causing major adverse events. However, no significant difference was observed in relapse-free survival, melanoma-related mortality, or overall survival between the treatment and placebo groups. These findings suggest that while high-dose vitamin D supplementation is safe and effectively raises serum vitamin D levels, it does not confer a clinical benefit in terms of melanoma recurrence or survival [56]. Another research favoured these findings, stating that they found a relevant linkage between vitamin D levels with the presence of CM and its depth [57]. More research is crucial to determine the safe dose of vitamin D. However, it is certain that its toxicity occurrence is low due to the self-regulating kidney mechanism. Therefore, maintain safety, it is advised to expose skin to UV radiation to achieve high levels of vitamin D [58]. A trial performed on Brazilian women showed that recommended supplementation of 15 μg/d cholecalciferol led to a healthy level of vitamin D in adult women, collectively preventing the increase in parathyroid hormone (PTH) [59]. A 2022 study confirmed significant seasonal fluctuations in 25(OH)D levels, with the lowest concentrations observed during autumn and winter, coinciding with UV exposure. Although an 8-week vitamin D supplementation regimen (5000 IU/day) temporarily elevated 25(OH)D levels, the effect was not sustained, indicating that continued supplementation may be necessary during periods of low sunlight. Seasonal changes in 25(OH)D were also accompanied by corresponding shifts in bone metabolism indicators, including calcium, phosphorus, and PTH, with unfavorable profiles observed during periods of vitamin D deficiency [60]. Studies show that vitamin D production increases linearly with UVR dose up to a point, after which it plateaus. Body surface area (BSA) exposure is positively but nonlinearly correlated with vitamin D synthesis, possibly due to anatomical differences in skin optics and 7-dehydrocholesterol (7-DHC) distribution [18,61]. The second International Conference on Controversies in Vitamin D concluded that serum vitamin D concentrations below 50 nmol/L are associated with negative health outcomes and currently affect approximately 25% of the global population [61,62]. To maintain adequate vitamin D levels through sun exposure, several factors must work together, such as latitude, skin type, season, and lifestyle. At higher latitudes, especially during winter, insufficient UV radiation makes it difficult to maintain vitamin D levels through sun exposure alone, particularly for individuals with darker skin types. In such cases, supplementation or dietary intake becomes necessary. During summer, longer sun exposure or greater skin surface area can help build vitamin D stores to offset winter declines [63,64]. A microsimulation study estimated the amount of sun exposure needed to maintain current 25(OH)D levels across Australia and New Zealand. The findings showed that in summer, 5–10 min outdoors on most days with 35% BSA exposed is sufficient. In contrast, during winter—especially at mid-to-high latitudes—longer midday exposure is required, often exceeding 45 min if only 10% of the body is exposed. These results can support the development of sun exposure guidelines and help healthcare providers identify individuals at risk of vitamin D deficiency [65]. Nevertheless, the complexity of vitamin D in its activity, healthy levels, and systemic effects still need further research. It would be advisable to take into consideration its duality with other, below-mentioned vitamins.

### 4.2. Vitamin A and Carotenoids

Vitamin A, a fat-soluble micronutrient, plays a pivotal role in maintaining skin homeostasis through its active derivatives, primarily retinoic acid and retinal. These metabolites regulate cellular proliferation, differentiation, apoptosis, and stem cell maintenance. Retinoic acid modulates hair follicle stem cell (HFSC) activity, melanocyte differentiation, and skin regeneration [66]. Carotenoids are plant pigments, divided into 2 groups, one of which is provitamin A. Vitamin A can be sourced either as preformed vitamin A from animal-derived foods or as provitamin A carotenoids found in fruits and vegetables. It is widely recognized for its beneficial biological roles, including photoprotection, immune system support, and regulation of oxidative stress. Additionally, both vitamin A and carotenoids have been shown to influence tumor cell behavior by modulating their proliferation, growth, and differentiation [67].

#### 4.2.1. Cellular Mechanisms of Action

Vitamin A exerts its biological effects through a tightly regulated metabolic pathway involving retinol, retinal, and retinoic acid. Circulating retinol is transported via RBP4 and transthyretin, entering target cells through STRA6 and RBPR2. Once intracellular, retinol is sequestered by esterification via LRAT or DGAT1 or oxidized to retinal by members of the SDR enzyme family such as RDH1, RDH10, and DHRS9. Retinal is then irreversibly converted to retinoic acid (RA) by ALDH1A isoforms, which bind CRABP1/2 to mediate nuclear receptor signaling via RARs and RXRs, or are degraded by CYP26 enzymes. Retinoic acid influences skin and hair physiology through nuclear transcriptional regulation and non-genomic pathways. It supports HFSC and melanocyte stem cell regulation, as well as melanocyte proliferation, and differentiation. Notably, UV exposure reduces cutaneous retinoid levels, potentially impairing melanogenesis and skin integrity. Furthermore, RA modulates epidermal proliferation through STRA6 and downstream JAK/STAT pathways, as well as through the FABP5-PPARβ/δ axis, contributing to its pleiotropic effects on cutaneous gene expression [66,67,68]. Moreover, vitamin A and carotenoids play crucial roles in counteracting oxidative stress through their potent antioxidant properties. During normal aerobic metabolism, ROS and RNS are generated, which can damage cellular components and contribute to the pathogenesis of various degenerative conditions, including skin cancers. Carotenoids, with their extensive conjugated double-bond systems, are particularly effective at quenching singlet oxygen (^1^O_2_) and neutralizing ROS and RNS through mechanisms such as electron transfer, hydrogen atom transfer, and radical adduct formation. Through these actions, vitamin A and carotenoids help preserve cellular integrity, modulate oxidative stress, and may exert protective effects against various malignancies [69,70]. A 2023 study shows that several dietary antioxidants—including vitamin C, β-carotene, and retinol—can accelerate malignant melanoma metastasis in vivo, likely through reducing ROS levels and increasing cell migration. While only a subset of antioxidants has this effect, the findings align with earlier studies and suggest caution in the use of antioxidant supplements by individuals at risk of cancer. These results also highlight the potential of targeting oxidative stress pathways, such as those involving the transcription factor BACH1, for future cancer therapies [71].

#### 4.2.2. Role in Skin Differentiation and Immune Modulation

Vitamin A plays a vital role in the immune system by regulating cytokine production, maintaining mucosal barrier integrity, and directing immune cell homing to mucosal surfaces. It supports the function and development of innate immune cells such as NK cells, macrophages, and neutrophils, and helps balance pro- and anti-inflammatory responses by influencing cytokines such as IL-10, IL-12, and TNF-α. Retinoic acid promotes the differentiation of regulatory T cells while suppressing proinflammatory Th1 and Th17 cells, helping to maintain immune tolerance. It also induces gut-homing markers (α4β7 and CCR9) on lymphocytes and B cells, thereby enhancing mucosal immunity and IgA production, which are essential for defending against intestinal pathogens [72,73]. Furthermore, retinoic acid (RA) plays a crucial role in melanocyte differentiation, with early exposure promotes melanocyte development from stem cells, whereas later or high-dose treatment impairs melanocyte maturation and function. In vivo, increased endogenous RA induces ectopic melanocyte differentiation via c-Kit signaling. In vitro, RA enhances melanogenesis in embryonic stem cells and melanoblasts, but suppresses it in mature melanocytes, where it inhibits proliferation, dendrite formation, and melanin production. While melanocytes can synthesize RA, expression of key RA-metabolizing enzymes such as LRAT, STRA6, and CRBP is variable, and the exact RA metabolic pathways active in melanocytes remain to be fully elucidated [66]. Summing up, vitamin A and carotenoid deficiency lead to impairment of the immune system and as a result, a higher risk of melanoma.

### 4.3. Vitamin C

#### 4.3.1. Sources and Metabolism

Vitamin C (ascorbic acid) is a water-soluble micronutrient that humans must receive from external sources, as they lack the enzyme L-gulono-γ-lactone oxidase required for endogenous synthesis [74]. Major dietary sources of vitamin C include citrus fruits, strawberries, kiwi, tomatoes, bell peppers, and green leafy vegetables, while animal-based sources contain relatively low concentrations [74]. Following oral intake, vitamin C is absorbed in the small intestine through the sodium-dependent vitamin C transporters (SVCT1 and SVCT2). SVCT1 is primarily responsible for intestinal absorption and renal reabsorption, while SVCT2 facilitates cellular uptake across various tissues, including the skin [74,75]. Bioavailability of vitamin C is tightly regulated and dose-dependent: intestinal absorption becomes saturated at high doses, limiting systemic levels. Plasma concentrations typically plateau at around 200 µmol/L in healthy individuals, whereas higher pharmacological levels can be achieved only by intravenous administration [76]. In the skin, vitamin C is actively accumulated from the bloodstream and reaches millimolar intracellular concentrations, particularly in the epidermis, which expresses both SVCT1 and SVCT2 transporters, which is unique among peripheral tissues [75]. This dual transporter expression suggests a high local demand for ascorbic acid in maintaining antioxidant defences and structural integrity. Notably, the epidermis exhibits 2–5 times higher f vitamin C concentrations than the dermis, and these levels decrease significantly with age, UV exposure, and environmental pollution [75]. Topical application of vitamin C has been explored to enhance cutaneous levels, especially when systemic delivery is insufficient. However, effectiveness is limited by poor dermal penetration due to the hydrophilic and charged nature of ascorbic acid. Stabilized derivatives and liposomal formulations have been developed to overcome this barrier, though their in vivo conversion to active vitamin C remains a subject of ongoing research [75]. Deficiency in vitamin C, even at subclinical levels, impairs collagen synthesis, reduces antioxidant protection, and compromises cutaneous immune competence. Thus, maintaining sufficient vitamin C intake is not only essential for systemic health but also crucial for preserving skin integrity and function, especially under oxidative or oncogenic stress [74,75,77].

#### 4.3.2. Antioxidant and Immunomodulatory Potential in Melanoma

Vitamin C is one of the most common exogenous vitamins, well known for its strong antioxidant properties. However, its biological role is much wider than direct free radical scavenging. Ascorbic acid plays a crucial part in maintaining intracellular redox homeostasis by regenerating other antioxidants such as glutathione, tocopherol (vitamin E), and thioredoxin, and by stimulating the expression and activity of endogenous antioxidant enzymes, including superoxide dismutase (SOD), catalase, and glutathione peroxidase. Moreover, it activates redox-sensitive transcription factors such as Nrf2, Ref-1, and AP-1, promoting the transcription of antioxidant and cytoprotective genes [78]. Through these mechanisms, vitamin C protects DNA, proteins, and lipids from oxidative damage. Under specific conditions—especially in the presence of free transition metals—it may also exert pro-oxidant activity via Fenton-type reactions [78]. Its dual redox behavior is therefore context-dependent and may be different between healthy and malignant cells. In the context of skin physiology, vitamin C also plays a role in regulating melanogenesis. Experimental models using UVA-irradiated human melanoma cells have shown that ascorbic acid suppresses melanin accumulation not by directly inhibiting tyrosinase, but by reducing UVA-induced oxidative stress and NO production. It achieves this through the restoration of catalase activity, enhancement of intracellular glutathione (GSH) levels, and downregulation of induced (iNOS) and endothelial (eNOS) nitric oxide synthases [79]. This suggests that the anti-melanogenic effect of vitamin C is largely mediated via modulation of redox-sensitive signaling pathways rather than enzymatic blockade. Interestingly, recent studies also highlight the potential pro-oxidant cytotoxicity of vitamin C in melanoma cells. Heavily pigmented melanoma cell lines with impaired antioxidant defenses, such as SK23, exhibit elevated basal ROS and DNA damage. In such cells, pharmacological doses of ascorbate significantly enhance hydrogen peroxide-induced strand breaks and clonogenic death—while sparing non-cancerous keratinocytes. This effect is further amplified by redox-active compounds such as Elesclomol, indicating that ascorbate may act as a selective enhancer of oxidative stress–based therapies in melanoma [80]. However, this dual role of vitamin C raises concerns. Recent in vivo research revealed that vitamin C supplementation at ROS-lowering doses may paradoxically promote melanoma metastasis. In NRAS- and BRAF-mutant melanoma models, ascorbate enhanced cell migration and invasion by activating the redox-responsive transcription factor BACH1. In mice models, oral administration of vitamin C doubled the number of metastases to lymph nodes and liver, without affecting primary tumor growth [72]. These findings suggest that while vitamin C may be protective in normal skin, it could potentially facilitate tumor progression under certain oncogenic and oxidative conditions, warranting caution in the context of high-risk individuals and melanoma patients. Beyond its antioxidant and redox-modulating roles, vitamin C also regulates innate immune functions. It has been shown to enhance the cytotoxic activity of natural killer (NK) cells, which play a key role in antitumor immunity. A recent study demonstrated that a stabilized form of vitamin C, known as Aptamin C, significantly increased NK cell activation and tumor-killing ability in vivo, primarily through activation of the STAT3 signaling pathway [81]. This immunoenhancing property may contribute to the antitumor potential of vitamin C, especially during the early immunosurveillance phase of melanoma prevention.

The dual role of vitamin C in different cellular contexts, highlighting its antioxidant, pro-oxidant, melanogenic, and immunomodulatory properties, as well as the associated clinical outcomes, is summarized in Table 1.

#### 4.3.3. Synergistic Effects with Other Antioxidants

Vitamin C interacts synergistically with other antioxidants, enhancing the overall capacity of cells to neutralize oxidative stress. Its best-known cooperation is with vitamin E (α-tocopherol). Vitamin E protects lipid membranes by scavenging lipid radicals, and vitamin C regenerates its active form from the α-tocopheroxyl radical. This interplay is supported by selenium-dependent enzymes such as glutathione peroxidase and thioredoxin reductase, forming a functional ascorbate-tocopherol-selenium axis that stabilizes cellular membranes, prevents propagation of lipid peroxidation, and maintains redox balance during oxidative stress [82]. Vitamin C also contributes to the redox recycling of thiol-based antioxidants such as ergothioneine, and although direct interaction with carotenoids is limited, its presence appears to enhance the protective effects of vitamin E in carotenoid-rich systems [83]. Importantly, recent studies show that this synergy is tissue specific. Vitamin C deficiency reduces vitamin E levels in the liver and heart, while vitamin E deficiency lowers vitamin C in the kidneys [84]. These findings highlight that vitamin C not only functions as a direct antioxidant but also stabilizes and recycles other antioxidants, especially under oxidative stress conditions relevant to skin and melanoma pathophysiology.

### 4.4. Vitamin E (Tocopherols and Tocotrienols)

Vitamin E, particularly in the form of α-tocopherol, is a potent lipophilic antioxidant known for its skin-protective properties and is widely used in cosmetics and supplements. It helps prevent oxidative damage, supports wound healing, and may alleviate symptoms of immune-mediated skin conditions. Clinical studies suggest that vitamin E supplementation, alone or in combination with other antioxidants, can improve skin condition and disease severity [85].

#### 4.4.1. Antioxidant Functions and Photoprotection

Vitamin E acts as an antioxidant that helps neutralize free radicals and may protect against melanoma by reducing oxidative stress and DNA damage. In vitro and animal studies suggest it can inhibit melanoma cell growth and UV-induced skin damage [86]. Vitamin E is particularly effective in protecting cell membranes from oxidative damage and inhibiting lipid peroxidation. It is also widely used to suppress LDL oxidation. The antioxidant strength of vitamin E depends on its chemical structure, especially the number of methyl groups on its chroman ring. Its activity is also influenced by its microenvironment, including lipid composition and the presence of other antioxidants [87]. Moreover, vitamin E demonstrates significant dermatological and anti-cancer benefits due to its antioxidant properties. Topical application of vitamin E enhances photoprotection against UVB radiation, promotes collagen synthesis, and inhibits collagen degradation, thereby contributing to anti-ageing effects. In vitro studies also suggest its role in suppressing melanin production via enhanced melanosome degradation. Combined antioxidant therapies, such as vitamin E with vitamin C or coenzyme Q10, have shown superior protection against oxidative stress in skin cells [88,89,90,91]. Beyond dermatology, vitamin E exhibits notable anti-cancer effects by inhibiting tumor cell proliferation, angiogenesis, and promoting apoptosis, through mechanisms involving NF-κB suppression. These findings support the therapeutic potential of vitamin E in skin health and oncology [92]. Furthermore, vitamin E is the most often occurring antioxidant in sunscreens. Its role is to help neutralize ROS generated by UVA radiation, thereby mitigating oxidative stress-induced skin damage and wrinkle formation. It contributes to the stabilization of UV filters, reducing the formation of harmful photo-oxidation by-products, and enhancing the overall photoprotective efficacy of sunscreens. Moreover, the inclusion of vitamin E in topical formulations has been shown to bolster the skin’s intrinsic defense systems and improve resistance to UV-induced erythema and immunosuppression [93]. Vitamin E isomers, particularly tocotrienols (TTs) have shown promising anticancer effects. Recent studies have demonstrated that TTs significantly reduce viability and induce apoptosis in melanoma cell lines without harming normal melanocytes, indicating selective cytotoxicity. This proapoptotic effect is mediated in part through activation of ER stress pathways, specifically the PERK/eIF2α/ATF4/CHOP and IRE1α branches, leading to intrinsic apoptosis. These findings suggest that TTs may represent a safe and effective candidate for novel chemopreventive or combinational therapies targeting melanoma [94]. Another study confirmed those findings by identifying a stem-like subpopulation in melanoma cells characterized by melanosphere formation, expression of stemness markers, and enhanced tumorigenic capacity. Notably, TTs effectively impaired cancer stem cells (CSCs) features, including melanosphere formation and ABCG2 expression, unlike standard therapy (vemurafenib), which spared CSCs. These findings suggest that TT selectively targets both the bulk tumor and CSC compartments, possibly by inhibiting ER stress and COX-2 pathways, supporting its potential as a safe and effective agent for overcoming melanoma resistance and recurrence [95].

#### 4.4.2. Dietary Intake and Supplementation

**Figure 2:**
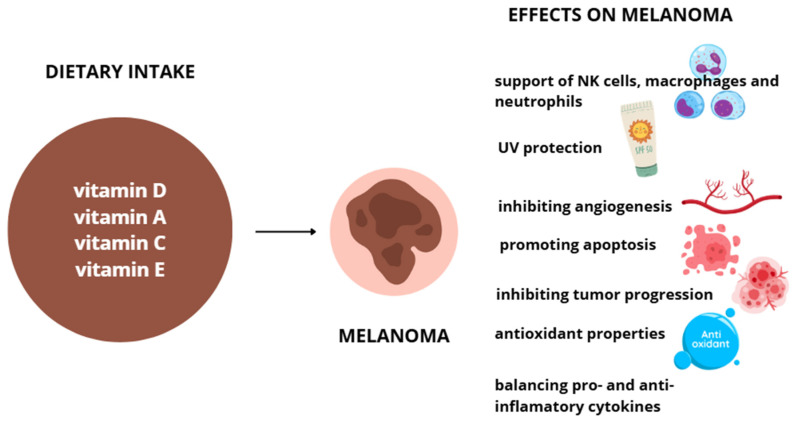
Effects of vitamins D, A, C, and E on melanoma cells.

Vitamin E is found in plant-based oils, nuts, seeds, fruits, and vegetables. Its content varies by plant part, variety, and processing methods, with wheat germ oil, sunflower oil, and nuts among the richest dietary sources [96]. The recommended daily intake ranges from 12–15 mg/day for healthy adults. While supplementation is generally safe, high doses may affect drug metabolism by inducing CYP3A4 enzymes, raising concerns about potential interactions with certain medications [97]. Symptoms of overdosing can vary, depending on the excessive amount of vitamins. Usually, in those cases bleeding, weakness, cardiovascular, and emotional problems occur. Effects of vitamin D, A, C and E supplementation on melanoma are shown on Figure 2.

### 4.5. Other Essential Vitamins: Evidence Gaps in Melanoma

Beyond vitamins A, C, E and D, other vitamins (particularly B-group vitamins involved in one-carbon metabolism and DNA methylation) have plausible links to carcinogenesis; however, melanoma-specific human data remain limited and inconsistent. For example, prospective cohort analyses reported mixed associations between one-carbon metabolism-related nutrients (including folate) and cutaneous melanoma risk [98]. Nicotinamide (vitamin B3) has demonstrated photoprotective and DNA repair-related effects in humans and reduced keratinocyte cancer incidence in high-risk populations, but evidence for melanoma endpoints is currently insufficient for strong conclusions [99,100].

## 5. Trace Elements and Melanoma Risk

### 5.1. Selenium

Selenium is an essential trace element involved in the synthesis of selenoproteins. These proteins exhibit a broad range of biological functions, including pleiotropic effects such as antioxidation and anti-inflammatory effects, mechanisms that critically contribute to carcinogenesis [101]. Glutathione peroxidase 1 (GPx1) is one of the most important cellular antioxidant enzymes, as it can be found in nearly all tissues of the body. In both the mitochondria and the cytoplasm, it modulates cellular ROS and maintains the balance between necessary and harmful levels of hydrogen peroxide. This limits the ROS-mediated DNA damage, which may otherwise promote cancer initiation. However, while GPx1 activity protects healthy cells against oxidative DNA damage, it may promote the survival of tumor cells. The increased activity of this protein may reduce the effectiveness of chemotherapy agents such as cisplatin by eliminating harmful ROS in tumor cells. The expression of the protein is strictly connected with the concentration of selenium. Low selenium levels lead to reduced translation of the GPx1 [102].

Selenium is also involved in the DNA repair process and has been shown to exhibit anticancer properties. This strongly suggests that it may play a role in the pathogenesis of skin-related cancers such as melanoma [101,103].

In this research, we found a meta-analysis examining the effect of Selenium on cancer prevention [4]. Out of three identified randomized controlled trials regarding melanoma, only two were included in the analysis due to low risk of bias. A statistically non-significant trend in which melanoma incidence increased with selenium supplementation was observed. The estimated summary relative risk (RR), for the effect of selenium supplementation on melanoma incidence, was 1.35 (95% CI 0.41 to 4.52). The authors suggested that there was no evidence of a positive effect of selenium supplementation in humans [104].

However, Rogoża-Janiszewska et al. conducted a prospective cohort study to assess whether serum selenium levels could be used as a prognostic biomarker for 10-year survival after a melanoma diagnosis. The group of 375 patients was divided into four categories according to serum selenium concentration. The subgroup with the highest selenium concentration > 96.15 µg/L had the most advantageous survival rate, whereas patients with selenium level < 76.23 µg/L demonstrated markedly increased mortality risk, with a hazard ratio (HR) of 8.42; *p* = 0.005 [105].

To assess the causal effect of selenium levels on melanoma risk and minimize confounding and reverse causation, Mendelian randomization was employed. The study showed that genetically predicted higher serum selenium levels were strongly associated with a reduced risk of malignant melanoma. The authors note that the results may pave the way for the development of potential biochemical targets for melanoma treatment [106]. However, under specific conditions, like high concentrations, selenium may promote melanoma cell growth and dissemination. The role of selenium in melanoma appears to be complex, and further research should be conducted to establish the optimal selenium dosage and its impact on melanoma stages [7].

Based on our research, we propose a potential dual role of selenium in cancer prevention and promotion. The role of selenium appears to be paradoxical, but it may be an important prognostic factor, and further research should be conducted.

### 5.2. Zinc

Zinc plays a fundamental role in the human body. Zinc ions are components of a wide range of proteins and are involved in multiple processes associated with carcinogenesis. They stabilize the DNA double helix and contribute to the formation of microtubules within the karyokinetic spindle. Zn ions are crucial for stabilizing the tumor suppressor protein p53 and its affinity for DNA. Furthermore, as a part of superoxide dismutase (SOD1, SOD3), which catalyzes the dismutation of superoxide anion radicals to hydrogen peroxide, zinc ions prevent the generation of other toxic free radicals and their derivatives. Zinc stabilizes zinc finger structures, which plays an important role in the regulation of DNA repair, transcription, and translation. Each of these mechanisms is essential for cell proliferation, differentiation, and apoptosis [107].

Zinc is also necessary for NK cells activation, and supplementation with this element significantly increases their number. These cells can recognize and eliminate melanoma cells. They are considered a positive factor in antitumor response [108].

Low zinc levels may lead to destabilization of the p53 protein, which leads to impaired apoptosis and uncontrolled proliferation. Moreover, insufficient levels of this trace element may increase the risk of cancer initiation by reducing the efficiency of DNA repair mechanisms. However, too high doses of zinc may lead to immunosuppression because of inhibition of lymphocyte function and INF-γ production [107].

Provinciali et al. conducted a study to examine the in vitro effect of zinc on the apoptosis of melanoma cells. Concentrations from 33.7 µM to 75 µM induced apoptosis in the human melanoma cell line WM 266-4. This effect was probably associated with the modulation of intracellular ROS levels and the associated induction of protein p53 [109].

Besides systemic action, zinc may also exert beneficial effects through external applications, including photoprotection. Zinc oxide (ZnO) plays a key role in this context. As a physical filter, it absorbs mainly UVA but also UVB radiation. Due to DNA damage, these agents are classified as carcinogens. They are strongly involved in the initiation and promotion of skin cancers, including melanoma. Regular use of sunscreens, especially during childhood, may greatly reduce the risk of developing melanoma [110].

Clinical data, mainly based on epidemiological studies, remain inconclusive and inconsistent. While some studies suggest elevated zinc levels in patients with melanoma, others report them to be reduced. The impact of zinc exposure is also uncertain, and available clinical evidence remains inconclusive [111].

Despite the numerous, well-described biological mechanisms through which zinc may affect melanoma initiation, progression, and therapy, its clinical relevance remains unclear. To access this information, further investigation is required.

### 5.3. Copper

Copper (Cu) is a trace element involved in numerous biological processes. Recently, increased attention has been paid to discovering its role in cancer biology. Copper has been observed to play a protumorigenic role in melanoma development. Copper influences many proteins and enzymes involved in tumor initiation. Tyrosinase, which is a rate-limiting enzyme in melanin biosynthesis, was identified as a copper-binding protein. The presence of copper enables the hydroxylation of l-tyrosine to 3,4-dihydroxyphenylalanine and oxidation to DOPAquinone. This indicates that copper is necessary to produce normal pigments in melanocytes and melanoma cells, allowing the melanoma cells to survive. Copper ions participate in the activation of the BRAF/MEK/ERK signalling pathway, in which mutations are found in more than half of melanomas. Moreover, elevated serum copper levels have been reported in cancer patients [112].

However, also antimelanoma effects of copper were observed. Excessive levels of copper have been associated with the induction of apoptosis, ferroptosis, and oxidative stress, which are responsible for paraptotic cell death. Recently, a new cell death mechanism, called cuproptosis, has been described. Cuproptosis is a programmed cell death that results from the excessive influx of copper ions into the cell from the environment. This makes cuproptosis a potential therapeutic target [112].

Wang et al. conduced an observational and Mendelian randomized study to investigate the association between copper intake and melanoma risk. Studies have shown that high dietary copper intake may be a protective factor against melanoma development. Dietary copper intake over 2.5 mg/day significantly reduces the risk. No causal relationship between serum copper and melanoma risk was observed. This may suggest that the underlying mechanism of the protective effect of higher copper intake is more complicated [113].

Despite well-described biological mechanisms, the role of copper in melanoma initiation remains unclear. Further investigation should be conducted.

Selenium, zinc, and copper may have a potentially dual effect on melanoma development. Despite exhibiting mechanisms that protect against the development of this cancer, in some situations they may promote melanoma progression or support the survival of cancer cells. This is summarized in Table 2.

### 5.4. Other Trace Elements

Apart from selenium, zinc, and copper, little is known about the role of other trace elements in melanoma development. Manganese is a component of mitochondrial superoxide dismutase (MnSOD), the overexpression of which is considered to have potential anti-cancer properties. However, studies have not demonstrated a significant correlation between urinary manganese concentration and melanoma development [114]. Iron plays a role in regulating ferroptosis, a form of controlled cell death. Its deregulation may promote the initiation and progression of cancer. However, a Mendelian randomization study has not confirmed a significant causal relationship between iron levels and melanoma development risk [7,115]. No significant studies evaluating the involvement of fluorine, iodine, and molybdenum in melanoma initiation have been published in recent years. This makes it impossible to draw reliable conclusions, indicating the need for further analysis.

All dietary sources of micronutrients, vitamin D, A, C, and E, and selenium, zinc, copper, are summarized in Table 3.

## 6. Other Dietary Bioactives Relevant to Melanoma Prevention

### 6.1. Omega-3 Polyunsaturated Fatty Acids (PUFAs)

Omega-3 polyunsaturated fatty acids (PUFAs) include alpha-linolenic acid (ALA), eicosapentaenoic acid (EPA) and docosahexaenoic acid (DHA); ALA is mainly derived from plant sources (e.g., flaxseed, chia, walnuts, canola oil), whereas EPA and DHA are predominantly obtained from marine sources (e.g., microalgae, fish oil, krill oil) [119]. Mechanistically, omega-3 PUFAs may modulate UV-related skin carcinogenesis through (i) remodeling of arachidonic-acid (AA) metabolism by competing for cyclooxygenase/lipoxygenase pathways and shifting eicosanoid profiles towards less pro-inflammatory species, and (ii) generation of specialized pro-resolving mediators (SPMs, e.g., resolvins, protectins, maresins) that actively promote resolution of inflammation [120,121]. In humans, an EPA supplementation trial demonstrated enrichment of EPA in skin lipids (reduced AA:EPA ratio) and measurable changes in cyclooxygenase- and lipooxygenase-derived eicosanoids under basal conditions and following a pro-inflammatory UV radiation challenge [120]. Translational evidence from randomized clinical studies also suggests potential photoprotective and immunomodulatory effects: an EPA-rich omega-3 intervention partially attenuated solar-simulated radiation-induced suppression of cutaneous cell-mediated immunity [122], and purified EPA (4 g/day, 3 months) increased the UV-induced erythemal threshold and reduced UV-induced p53 expression in skin, indicating attenuation of early UV-related genotoxic responses [123]. Nevertheless, recent systematic reviews emphasize that supplement trials in photoprotection remain heterogeneous and often small, with mixed outcomes across endpoints [124]. Importantly, evidence based on melanoma incidence or clinical endpoints is still insufficient, and Mendelian randomization findings are not fully concordant; notably, a recent two-sample MR reported higher genetically proxied serum omega-3 levels associated with increased risks of basal cell carcinoma, squamous cell carcinoma and melanoma, with sensitivity to variants in endogenous PUFA metabolism (e.g., FADS1) [125].

### 6.2. Polyphenols

Polyphenols are a large and chemically diverse group of compounds that occur in many fruits, vegetables, and other dietary sources. Available evidence suggests that diets rich in polyphenol-containing foods may be associated with a reduced incidence of several cancers [126]. Due to their antioxidant, anti-inflammatory, and antiproliferative activities, polyphenols have also been proposed as complementary agents in melanoma prevention. Mechanistically, several polyphenols (e.g., curcumin, resveratrol, quercetin and others) have been reported to modulate key melanoma-related signaling pathways, including the mitogen-activated protein kinase (MAPK) pathway, phatidylinositol-3-kinase/protein kinase B (PI3K/Akt) signaling, cyclin-dependent kinase (CDK) and the Wnt pathway. In addition, polyphenols have been reported to affect transcriptional regulators involved in melanomagenesis, such as signal transducer and activator of transcription 3 (STAT3) and NFκB. These pathway effects converge on key cellular outcomes, including cell-cycle regulation, modulation of anti-apoptotic proteins, such as Bcl-2, and induction of apoptosis through p53-dependent mechanisms and caspase activation. Collectively, these effects are consistent with reduced melanoma growth in preclinical models [127].

Bridging these mechanistic insights into melanoma prevention in humans is not straightforward, and direct clinical evidence remains limited. However, in a human nutritional intervention study [128], ten healthy volunteers received 4–6 g/day of a cocoa powder rich in polyphenols for one week. This led to increased UV tolerance and reduced objective erythema following solar simulated irradiation. Although this intervention does not directly demonstrate the role of polyphenols in melanoma prevention, these findings suggest that dietary polyphenols may contribute to cutaneous photoprotection [128].

Additional clinical evidence comes from a randomized, double-blind, placebo-controlled clinical trial in healthy women (*n* = 65), in which participants received apple polyphenols (300 or 600 mg/day) for 12 weeks. The study found that continuous supplementation significantly improved UV irradiation induced skin responses, including reduced skin pigmentation compared with placebo [129].

Despite many proposed mechanistic pathways and emerging evidence from human studies suggesting positive effects of polyphenol rich interventions on cutaneous photoprotection and UV-response endpoints, well-designed randomized controlled trials are still needed to conclusively determine whether these compounds reduce melanoma risk and incidence.

## 7. Clinical and Public Health Implications

### 7.1. Potential for Micronutrient-Based Preventive Strategies

Current evidence suggests that select micronutrients, especially vitamins D, A, C, and E, zinc, selenium, and copper, may support endogenous antioxidant systems, reduce UV-induced oxidative stress, and modulate immune responses relevant to melanoma pathogenesis [30,73,79,89,113]. These effects, demonstrated in vitro, animal, and human studies, position micronutrients as potential adjuncts in skin cancer prevention, particularly in reducing oxidative DNA damage and supporting melanocyte homeostasis. These antioxidant-driven mechanisms act upstream of DNA repair processes and may reduce mutagenic burden in melanocytes, as highlighted in Section 4. Although no micronutrient can substitute for UV protection, nutritional interventions may complement behavioral and dermatologic strategies in integrated skin health programs.

### 7.2. Considerations for High-Risk Populations

Populations at increased risk of melanoma—such as fair-skinned individuals [12], people with high UV exposure, family history of melanoma, or certain genetic profiles—may derive particular benefit from optimizing micronutrient status relevant to redox balance and oxidative-stress defense pathways [13,130]. Importantly, genetic predispositions (e.g., SLC23A1, VDR polymorphisms) may significantly modify individual responsiveness to micronutrient supplementation, requiring risk stratification in high-UV and melanoma-prone populations. For example, transport polymorphisms in SLC23A1/2 may impair vitamin C bioavailability despite adequate intake, while VDR variants may influence the cutaneous and systemic actions of vitamin D. This highlights the potential utility of personalized micronutrient recommendations guided by nutrigenomic screening [30,38,54,131].

### 7.3. Caution in Supplementation

Despite promising results, supplementation must be approached with caution. Some antioxidants, including vitamin C, exhibit context-dependent effects: while protective in normal tissue, they may enhance tumor survival or metastasis under certain conditions, particularly in already-initiated tumors with altered redox signaling. Doses, timing, and combinations of micronutrients are critical factors. High-dose antioxidant supplementation should not be recommended indiscriminately, especially in individuals with existing neoplasms or high oncogenic risk. Current guidelines support a food-first approach, with supplements used selectively and based on evidence [72,81,82,129,132].

Selenium exemplifies this complexity. While essential for antioxidant defence via selenoproteins like GPx1, high enzymatic activity may also promote tumor cell survival and reduce the efficacy of certain chemotherapies. Although one prospective study suggested better melanoma prognosis at higher selenium levels, randomized trials failed to confirm a preventive effect, and under certain conditions, selenium may even support melanoma progression [7,106]. Similarly, zinc, though essential for immune and enzymatic functions, can impair copper metabolism and provoke redox imbalance when administered in excess [133].

### 7.4. Integration into Broader Preventive Frameworks

The priorities outlined in the International Agency for Research on Cancer (IARC) 2024 roadmap “Putting an end to cancer before it can begin” underline three mutually reinforcing pillars for cancer control: (I.) population-wide primary prevention, (II.) early detection to improve curability, and (III.) health-system interventions adapted to national resources [134].

Translating these pillars to melanoma means that classical UV-protection campaigns should be complemented by nutritional and micronutrient-based strategies that mitigate oxidative damage and support immune surveillance. In practical terms:

Primary prevention—balanced diets rich in vitamins D, A, C, and E plus trace elements such as selenium and zinc can lower the ROS activity that drives UV-induced mutagenesis, while avoiding high-dose supplementation that may paradoxically enhance tumour survival.

Early detection—educating high-risk groups (fair skin, family history, occupational UV exposure) to recognise suspicious lesions and to discuss micronutrient deficiencies with healthcare providers fits the IARC call for “screening-ready populations”.

Tertiary/survivorship care—for melanoma survivors, maintaining adequate micronutrient status may help reduce recurrence risk and improve quality of life by modulating chronic inflammation and supporting DNA-repair pathways.

In line with IARC’s pressure on evidence-based actions, any supplementation programme should be preceded by dietary assessment and, where possible, laboratory confirmation of deficiency. This ensures the efficient allocation of public health resources and minimizes the risk of adverse effects from excessive dosing.

## 8. Risk, Limitations and Future Directions

Although there are links between micronutrients and pathways relevant to melanoma development, recommendations for supplementation in patients should be treated with caution [12,13,132]. Supplementation should be limited to cases where deficiencies of a given component have been documented, or where there are other clearly justified reasons for doing so [19,135]. Initially, a varied, wholesome diet is needed [6]. It is crucial to emphasize the safety of supplementation. In the case of vitamin D, excessive oral supplementation may lead to hypercalcemia, which can cause gastrointestinal complications such as vomiting, nausea, and constipation, as well as renal complications. In severe cases, it can even cause kidney failure. Therefore, recommendations should be based on clinical data such as plasma 25(OH)D concentration and dietary habits to develop a safe strategy for possible supplementation [19,64]. Similar caution should be exercised regarding other micronutrients. For example, high doses of vitamin E (particularly α-tocopherol) can interfere with drug metabolism by increasing the expression of CYP3A proteins, which belong to the CYP3A4 family and are involved in the metabolism of many drugs [97]. Selenium supplements also pose a risk due to their narrow safety margin. Deficiency and excess can both be detrimental to health, and the upper limits of safe doses vary [103]. Excessive zinc intake can cause copper deficiency in patients, leading to anemia and neurological complications [133].

It is also important to note the heterogeneity of available supplements. These differences relate to recommended doses, dosage regimens, and the duration of supplementation. Furthermore, a study on selenium found inconsistencies between the declared and actual selenium content of supplements. All this makes it difficult to compare supplements and provide clear recommendations for patients [136,137,138]. It is also important to compare supplementation with dietary intervention and to choose the appropriate approach [139]. Therefore, further research is needed, especially in the form of large, prospective, randomized, controlled clinical trials with clinically relevant endpoints and harmonized dosing regimens. In addition, studying genetic factors that may influence nutritional status and response to supplementation could facilitate the formulation of more personalized recommendations.

## 9. Summary and Conclusions

Based on the analyses, it can be concluded that certain vitamins (D, A, C, and E) and trace elements, most notably selenium, zinc, and copper, play a significant role in modulating biological processes relevant to the pathogenesis and prevention of melanoma. These vitamins have been shown to possess antioxidant, immunomodulatory, and UV-induced DNA damage repair properties. Of particular importance is vitamin D deficiency, which has been shown to increase the risk of melanoma development and worsen the prognosis of patients.

Conversely, certain substances, including selenium and vitamin C, exhibit a biphasic effect, indicating that their capacity to elicit anti-tumor processes is depends on both the dosage and the biological state of the organism. Under specific conditions, these substances can also promote tumor progression. Furthermore, observations of vitamin D receptor (VDR) polymorphisms and vitamin C transporter genes suggest that supplementation efficacy may depend on genetic factors, thereby opening the prospect for personalized prevention.

Currently, there is no clear evidence that polyphenols or omega-3 reduce the risk or incidence of melanoma, and some genetic (MR) results remain inconsistent, justifying the need for larger, well-designed RCTs with clinical endpoints.

Despite the abundance of experimental and epidemiological data, there is still a lack of clear clinical evidence supporting the efficacy of supplementation as a stand-alone melanoma prevention strategy. Presently, the most significant elements of prevention remain reducing ultraviolet (UV) exposure and using photoprotection. Micronutrient supplementation should be regarded as a complementary measure, particularly for individuals who are at risk or have been diagnosed with deficiencies.

Further studies must be conducted to consider genetic variation. These studies will provide clear recommendations on the use of vitamins and trace elements in the prevention of melanoma.

## Figures and Tables

**Table 1:** Comparative overview of vitamin C effects in healthy skin cells and melanoma cells.

Biological Mechanism	Healthy Skin Cells	Melanoma Cells
Reduction in ROS	Efficient neutralization of oxidative stress	May also protect tumor cells from ROS damage
Activation of antioxidant enzymes (SOD, catalase, GPx)	Upregulation of endogenous antioxidant defense	Often compromised or dysregulated in cancer cells
Pro-oxidant activity	Rare: Fenton-type reactions under metal excess	Frequent: promotes oxidative DNA damage and apoptosis
Effect on melanogenesis	Reduces melanin via NO and ROS suppression	Reduces melanin indirectly; no direct inhibition of tyrosinase
Immunomodulatory effect (NK cells)	Enhances NK cell activation and cytotoxicity	May promote immunosurveillance in early tumor stages

**Table 2:** The dual action of zinc, selenium and copper in melanoma development.

Trace Element	Potential Anticarcinogenic Activity	Potential Procarcinogenic Activity
Selenium (Se)	Antioxidant/anti-inflammatory effects via selenoproteins Involvement in DNA repair [101,103]	Increased activity of GPx1 may promote survival of melanoma cells by reducing harmful ROS in tumor cells [102]
Zinc (Zn)	Stabilizing the tumor suppressor protein p53 and its affinity for DNA [107] Necessary for activation of NK cells, responsible for recognizing and eliminating cancer cells [108]	Excessive doses of zinc inhibit lymphocyte function and INF-γ production, leading to immunosuppression [107]
Copper (Cu)	Induction of a cell death mechanism called cuproptosis [112]	Cofactor for tyrosinase (melanogenesis)—supporting melanoma cell survival Supports pro-tumorigenic signaling (BRAF/MEK/ERK) [112]

**Table 3:** Dietary sources of vitamin D, A, C and E, and selenium, zinc, and cooper.

Micronutrients	Main Dietary Sources	References
Vitamin D	fish, beef liver, egg yolk, cheese	[16]
Vitamin A	fruits, cooked vegetables, red palm oil, olive oil	[67]
Vitamin C	citrus fruits, strawberries, kiwi, tomatoes, bell peppers, green leafy vegetables	[74]
Vitamin E	plant-based oils, nuts, seeds, fruits, vegetables	[96]
Selenium	fish, meat, milk and dairy products, eggs, cruciferous vegetables, onions, garlic	[116]
Zinc	meat, poultry, dairy products, sea food	[117]
Copper	milk and dairy products, meat and offal, cereal products, potatoes, carrot, broccoli, cabbage, seafood, apples, bananas	[118]

## Data Availability

Data is contained within the article, The original contributions presented in this study are included in the article. Further inquiries can be directed to the corresponding authors.

## Abbreviations

The following abbreviations are used in this manuscript:

^1^O_2_	Singlet oxygen
1,25(OH)_2_D	1,25-dihydroxyvitamin D
25(OH)D	25-hydroxyvitamin D
7-DHC	7-dehydrocholesterol
AA	Arachidonic acid
AhR	Aryl hydrocarbon receptor
ALA	Alpha-linolenic acid
AP-1	Activator protein-1
APCs	Antigen-presenting cells
cAMP	Cyclic adenosine monophosphate
CDK	Cyclin-dependent kinase
CM	Cutaneous melanoma
CPDs	Cyclobutane pyrimidine dimers
CSC	Cancer stem cells
Cu	Copper
CYP24A1	24-hydroxylase
CYP27B1	1α-hydroxylase
DAG	Diacylglycerol
DHA	Docosaheksaenoic acid
ECM	Extracellular matrix
eNOS	Endothelial nitric oxide synthase
EPA	Eicosapentaenoic acid
FADS1	Fatty acid desaturase 1
Fe	Iron
FGF23	Fibroblast growth factor 23
GPx1	Glutathione peroxidase 1
GSH	Glutathione
GxE	Gene–environment interactions
HFSC	Hair follicle stem cell
HR	Hazard ratio
H_2_O_2_	Hydrogen peroxide
IFN-γ	Interferon-gamma
IARC	International Agency for Research on Cancer
iNOS	Induced nitric oxide synthase
IP_3_	Inositol trisphosphate
LL-37	Cathelicidin
LXRα/β	Liver X receptors alpha/beta
MAPK	Mitogen-activated protein kinase
MHC	Major histocompatibility complex
miRNA	MicroRNA
Mn	Manganese
MnSOD	Manganese superoxide dismutase
NFκB	Nuclear factor kappa B
NLR	Neutrophil-to-lymphocyte ratio
NMSC	Non-melanoma skin cancers
NO	Nitric oxide
NER	Nucleotide excision repair
NK	Natural killer
Nrf2	Nuclear factor erythroid 2–related factor 2
O_2_•^−^	Superoxide anion
ORR	Objective response rate
PI3K/Akt	Phatidylinositol-3-kinase/protein kinase B
PTH	Parathyroid hormone
PPARγ	Peroxisome proliferator-activated receptor gamma
PUFAs	Polyunsaturated fatty acids
RA	Retinoic acid
RNS	Reactive nitrogen species
RORα/γ	Retinoic acid-related orphan receptors alpha/gamma
ROS	Reactive oxygen species
Se	Selenium
SOD	Superoxide dismutase
SPM	Specialized pro-resolving mediator
STAT3	Signal transducer and activator of transcription 3
SVCT	Sodium-dependent vitamin C transporter
TLR	Toll-like receptor
TTs	Tocotrienols
UV	Ultraviolet
UVB	Ultraviolet B
VDBP	Vitamin D-binding protein
VDR	Vitamin D receptor
VDREs	Vitamin D response elements
Vitamin C	Ascorbic acid
Vitamin D2	Ergocalciferol
Vitamin D3	Cholecalciferol
Vitamin E	Tocopherol
Zn	Zinc
ZnO	Zinc oxide
•OH	Hydroxyl radicals
